# Morphometric Analysis of the Cervical Canal Using Computed Tomography Scan Among Patients With Neck Pain in North India

**DOI:** 10.7759/cureus.25466

**Published:** 2022-05-29

**Authors:** Kanhaiya Jee, Yogesh Yadav, Nisha V Kaul, Harshita Pant

**Affiliations:** 1 Anatomy, Mayo Institute of Medical Sciences, Barabanki, IND; 2 Anatomy, Santosh Medical College and Hospital, Santosh Deemed to Be University, Ghaziabad, IND; 3 Radiodiagnosis, Mayo Institute of Medical Sciences, Barabanki, IND

**Keywords:** spinal canal, transverse foramen, spinal cord injury, facet joint osteoarthritis, cervical spinal stenosis

## Abstract

Introduction

Cervical spinal stenosis is a common disease that results in considerable morbidity and disability. To avoid long-term disability caused by irreversible spinal cord damage, quick diagnosis and treatment are required. To our knowledge, until recently, there has been no report or study evaluating the cervical canal stenosis and associated facet joint arthrosis as the major cause of neck pain, so the current study used computed tomography (CT) scans to determine the prevalence of cervical canal stenosis and facet joint osteoarthrosis in patients who presented with neck pain, including its relationship with age, sex, and cervical spinal levels (C3-C7).

Methods

The current clinical descriptive cross-sectional study was conducted in the Department of Anatomy and Radiodiagnosis at Santosh Medical College, Ghaziabad, for a period of 24 months among newly diagnosed outpatient department (OPD) cases of neck pain (18 years or older) with suspected cervical canal stenosis and facet joint arthrosis. Clinical history, patient-specific clinical examination, and relevant information were obtained in a structured data collection schedule through interviews during OPD hours. All of the participants underwent a CT scan of the cervical region. The independent factors (age, gender, height, and weight) were used in a multiple linear regression analysis of neck pain grading, Torg ratio (TR), and right and left facet joint degeneration, which were expressed as R-squared (R^2^) and adjusted R-squared (aR^2^). Statistical tests were executed at a 5% level of significance; an association was considered significant if the p-value was <0.05.

Results

A total of 83 subjects were enrolled in this study with equal representation from both sexes, i.e., males (49.4%) and females (50.6%). The transverse vertebral canal (T-VC) diameter was narrowest at the level of C3 (25.00 ± 1.13) and gradually increased at the level of C6 (25.18 ± 1.14) in this study. The mean TR of cervical vertebrae C3-C4 dropped gradually from C3 (0.78 ± 0.05) to C7 (0.76 ± 0.05) in this study. Severe left and right facet joint degeneration were observed in 13.3% and 10.5% of study subjects, respectively. In almost every subject, neck pain was a neurological symptom, so multiple linear regression analysis of neck pain grading was carried out with the independent variables (age, gender, height, and weight) and it was found to be not significant (R^2^ = 0.0617, aR^2^ = 0.0136, p = 0.2842).

Conclusion

The articulations of the posterior arch of the vertebrae are known as facet joints. They are a vital component of the vertebral column's structural stability. The superior and inner articular facets of the vertebrae are joined by these joints, which are encased in a fibrous capsule.

## Introduction

Cervical spinal stenosis is a common disease that results in considerable morbidity and disability [1]. To avoid long-term disability caused by irreversible spinal cord damage, quick diagnosis and treatment are required. Cervical vertebrae have smaller bodies than other vertebrae, and their purpose is to protect the spinal cord, support the head, and enable movement of the head in flexion, extension, and rotation [2].

It is estimated that 24.4% of the population suffers from spinal cord compression, which affects the cervical cord in 10% of cases [3]. Tumors, infections, trauma, and degenerative changes such as intervertebral disc herniation, osteophytes, and ossification of the posterior longitudinal ligaments can all cause the cervical canal to narrow [4]. Pain is the most prevalent presenting symptom, followed by numbness, tingling, weakness, gait instability, bowel and bladder dysfunction, spasticity and paresthesia, and, in rare cases, irreversible paraplegia. These variables may cause increasing cord compression, culminating in spinal cord ischemia, and histopathologic alterations in the cervical spinal cord [5,6].

The facet joints, also known as the zygapophyseal joints, are affected by osteoarthritis (OA) of the spine. In humans, the only functional synovial joints between neighboring spinal levels are paired diarthrodial joints (DJ) in the vertebral column's (VC) posterior section. When it comes to facet joint osteoarthritis (FJOA), it is important to remember that it is closely linked to the separate but functionally similar illness known as degenerative disc disease, which affects components in the anterior section of the spinal column. It is believed that both FJOA and degenerative disc disease are widespread causes of back and neck pain, which in effect get a significant influence on the healthcare systems and economy of developed nations [6].

FJOA is a clinicopathological phenomenon that is characterized by the dysfunction of synovial facet joints. The process of failure, although commonly thought of as a disorder characterized by loss of articular cartilages and hypertrophied bones, actually affects entire joints, along with soft tissue, periarticular paraspinal muscle, subchondral cartilages, bones and ligament, the capsule, and the synovium. The facet joint is part of a "motion segment" in the spine that also comprises the intervertebral discs, which degenerate in tandem with the facet joints. As a result, FJOA is usually linked to degenerative disc degeneration (DDD) [7].

At some time in their lives, the majority of adults will have neck discomfort that radiates to their upper limbs. Cervical spinal canal stenosis is a common cause of this issue. This disorder is characterized by a narrowing of the cervical spinal canal within the VC, which includes the spinal cord and its surrounding meninges, blood vessels, and spinal nerve roots [8]. This stenosis has long been associated with cervical spondylotic myelopathy and cervical neuropraxia associated with trauma, degeneration, and inflammation [9]. According to one study, 82% of persons aged 54 years and above show radiologic indications of cervical spine degeneration [10].

All of the spinal compression force, which rises in magnitude from the axis vertebra to the lumbosacral joint, is sustained by the vertebral bodies and intervertebral discs [11]. Narrowing of the sagittal diameter of the cervical canal in the adult spine is a causative factor, measured radiologically by X-ray [12]. The zygapophyseal or facet joints are the most important structures in determining the biomechanical properties of the cervical spinal column [13]. The foramen of the vertebrae creates the spinal canal, which is a hollow tunnel through which the spinal cord flows. The narrowing of the spinal canal, known as stenosis, has been linked to neurological damage.

To our knowledge, until recently, there has been no report or study evaluating the cervical canal stenosis and associated facet joint arthrosis as the major cause of neck pain, so the current study used computed tomography (CT) scans to determine the prevalence of cervical canal stenosis and facet joint osteoarthrosis in patients who presented with neck pain, including its relationship with age, sex, and cervical spinal levels (C3-C7).

## Materials and methods

Study setting and design

After receiving ethical approval from Santosh Medical College Ethics Committee, Ghaziabad (IEC/IRB No.: SMC/2019/2131(7)), the current clinical descriptive cross-sectional study was conducted for a period of 24 months (June 2019 to June 2021) in the Department of Anatomy and Radiodiagnosis at Santosh Medical College (SMC), Ghaziabad and Mayo Institute of Medical Sciences (MIMS), Barabanki, Uttar Pradesh.

Study subjects and sample size

The newly diagnosed cases of neck pain (18 years or above) with suspected cervical canal stenosis and facet joint arthrosis (paresthesia associated with movement of the neck, loss of sensibility corresponding to a dermatome, muscular weakness, atrophy of muscles, and rigidity of neck) from the outpatient department (OPD) of orthopedics and neurology of SMC and MIMS referred to the radiodiagnosis department of SMC and MIMS for CT scan were included in the present study as subjects. The minimum sample size was calculated as 72 considering the prevalence rate of the cervical canal stenosis as 4.9% and taking absolute precision as 5% [13]. Prior to enrolling subjects in the study, written informed consent was obtained from either the patient or relatives after a detailed explanation of the study's purpose, and a consecutive sampling method was used to enroll the study subjects, resulting in a total of 83 patients being enrolled in the study over the course of the study's defined duration. Patients with previous trauma (head or spine), surgery of the head or spine, psychiatric disorder, drug abuse, tumor of the head and neck, pregnancy, neoplasia, or spinal canal-associated congenital anomaly were excluded from the study.

Data collection and CT scan

Clinical history, patient-specific clinical examination, and relevant information were obtained in a structured data collection schedule through interviews during OPD hours. CT scans of the cervical region were performed for all the enrolled subjects using Somatom Scope G-XL-91368, version CTVC30, CT scan machine (Siemens, Munich, Germany; number of slices: 16), and CT scan images were interpreted by at least one senior radiologist who had experience in reporting of CT scans. The cervical vertebra in consideration was from C3 to C7. C1 and C2 were not included because of their different shapes compared with the other cervical vertebra.

Anthropometric measurements

Seca digital weight machine (Hamburg, Germany) was used to weigh the subjects with 0.1 kg precision. The subject's height was measured with a 0.1 cm measurement tape attached to the wall. The BMI was computed by dividing the body in kilograms by height in meters squared.

Statistical analysis

The data were collected and entered into a Microsoft Excel spreadsheet (Microsoft, Redmond, WA) and analyzed using the Statistical Package for Social Sciences (SPSS) version 26 software (IBM Corp., Armonk, NY). The demographics, clinical signs and symptoms, and CT scan radiological findings of patients were used to analyze the results. Continuous variables were reported as mean and SD, whereas categorical variables were presented as numbers and percentages (%). The Kolmogorov-Smirnov test was used to determine whether the data were normal. The non-parametric test was employed if normality was refused. The linear relationship of transverse vertebral canal (T-VC) diameter and Torg ratio (TR) among C3 to C7 vertebrae were analyzed using the correlation matrix analysis and an independent t-test was used to observe the difference in the mean values of T-VC and TG among C3-C7 vertebrae. Multiple linear regression analysis of neck pain grading, TR, and right and left facet joint degeneration was carried out with the independent variables (age, gender, height, and weight) and represented R-squared (R^2^) and adjusted R-squared (aR^2^). All tests were done at a 5% level of significance; an association was considered significant if the p-value was <0.05.

## Results

A total of 83 subjects were enrolled in this study with equal representation from both sexes, i.e., male (41/83, 49.4%) and female (42/83, 50.6%). The minimum age of study subjects was 40 years whereas the maximum age was 74 years (Table 1). Nearly two-fifth of subjects (43.3%) belonged to the age group of 51-60 years and only 3.6% of subjects were above 70 years of age. The BMI of the subjects ranged between 16 and 33 kg/m2. As per BMI classification, one-fourth of subjects were overweight (26.4%) and 4.8% of subjects were obese. In almost every subject, neck pain was a neurological symptom, so multiple linear regression analysis of neck pain grading was carried out with the independent variables (age, gender, height, and weight) and it was found to be not significant (R^2^ = 0.0617, aR^2^ = 0.0136, p = 0.2842).

**Table 1:** Baseline characteristics of study subjects (N = 83).

Variable	Number (%)/mean ± SD
Gender	
Male	41 (49.4)
Female	42 (50.6)
Age (years)	57.98 ± 7.63
Age groups (years)	
40-50	14 (16.8)
51-60	36 (43.3)
61-70	30 (36.1)
>70	3 (3.6)
Height (cm)	167.18 ± 8.37
Weight (kg)	67.47 ± 9.71
Body mass index (BMI) (kg/m^2^)	24.20 ± 3.72
BMI classification	
Healthy weight	43 (51.8)
Underweight	5 (6.0)
Overweight	22 (26.4)
Obese	4 (4.8)
Neurologic symptoms	
Neck pain	82 (98.8)
Numbness	44 (53.0)
Tingling	21 (25.3)
Weakness	6 (7.2)
Gait instability	5 (6.0)

The mean T-VC diameter for C3, C4, C5, C6, and C7 was 25.00 ± 1.13 mm, 25.10 ± 1.13 mm, 25.09 ± 1.13 mm, 25.18 ± 1.14 mm, and 24.39 ± 1.12 mm, respectively. The TR for C3, C4, C5, C6, and C7 was 0.78 ± 0.05, 0.76 ± 0.05, 0.75 ± 0.05, 0.76 ± 0.05, and 0.76 ± 0.05, respectively (Table 2).

**Table 2:** Transverse vertebral canal diameter and Torg ratio of study subjects (N = 83).

Vertebrae	Transverse vertebral canal diameter in mm (T-VC), mean ± SD (range)	Torg ratio, mean ± SD (range)
C3	25.00 ± 1.13 (22.35-27.67)	0.78 ± 0.05 (0.66-0.91)
C4	25.10 ± 1.13 (22.47-27.79)	0.76 ± 0.05 (0.64-0.88)
C5	25.09 ± 1.13 (22.44-27.76)	0.75 ± 0.05 (0.64-0.87)
C6	25.18 ± 1.14 (22.53-27.85)	0.76 ± 0.05 (0.65-0.88)
C7	24.39 ± 1.12 (21.74-27.06)	0.76 ± 0.05 (0.64-0.88)

The linear relationship of T-VC diameter among C3-C7 vertebrae was analyzed and the correlation matrix showed that the linear relationship was statistically significant between most of the vertebrae except between vertebrae C3 and C4, C4 and C5, and C4 and C6 (Table 3). Similarly, the linear relationship of TRs among C3-C7 vertebrae was analyzed and the correlation matrix showed that the linear relationship was statistically significant between most of the vertebrae except between vertebrae C6 and C7, but multiple linear regression analysis of TRs for C3-C7 vertebrae was carried out with the independent variables (age, gender, height, and weight) and it was found to be not significant for any of the vertebrae (C3: R^2^ = 0.0602, aR^2^ = 0.012, p = 0.2974; C4: R^2^ = 0.0683, aR^2^ = 0.0206, p = 0.2318; C5: R^2^ = 0.065, aR^2^ = 0.017, p = 0.2574; C6: R^2^ = 0.0656, aR^2^ = 0.0177, p = 0.2526; and C7: R^2^ = 0.0614, aR^2^ = 0.0133, p = 0.2866).

**Table 3:** Characteristics of left and right facet joint degeneration among study subjects (N = 83).

Criteria	Left facet joint degeneration, number (%)	Right facet joint degeneration, number (%)
Joint space narrowing		
Yes	42 (50.6)	40 (48.2)
No	41 (49.4)	43 (51.8)
Osteophytes		
Yes	32 (38.5)	30 (36.1)
No	51 (61.5)	53 (63.9)
Irregularity of articular surface		
Yes	41 (49.4)	38 (45.8)
No	42 (50.6)	45 (54.2)
Overall degree of facet joint degeneration		
0	21 (25.3)	21 (25.3)
1	20 (24.0)	25 (30.1)
2	31 (37.3)	28 (33.7)
3	11 (13.3)	09 (10.5)
Severity		
Mild	20 (24.0)	25 (30.1)
Moderate	31 (37.3)	28 (33.7)
Severe	11 (13.3)	09 (10.5)

**Figure 1:**
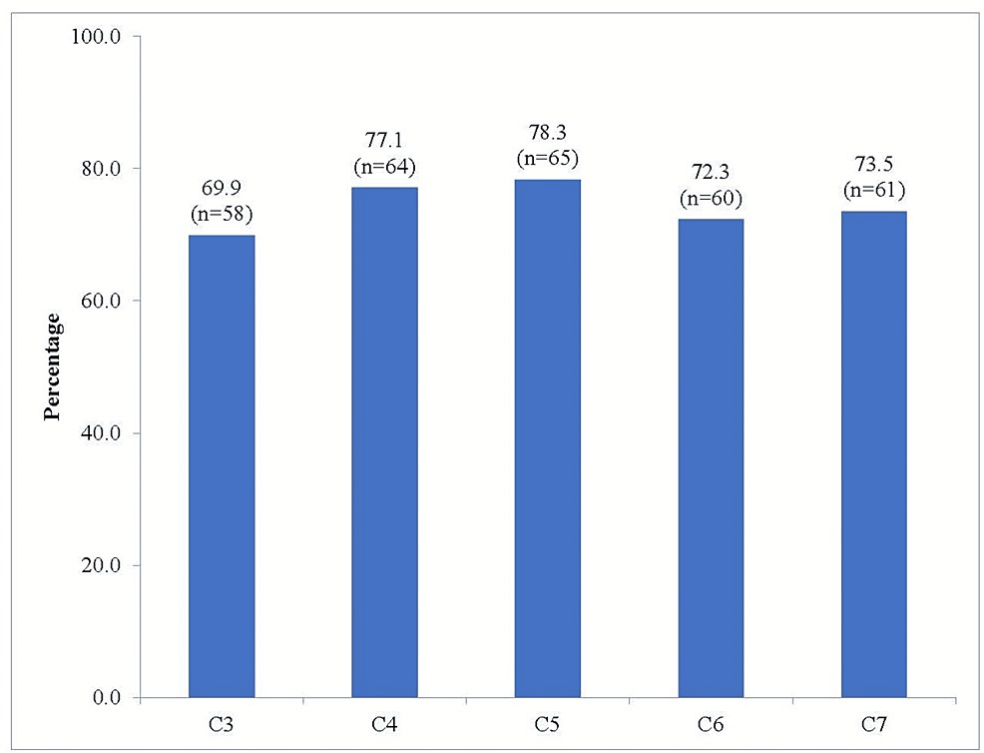
Clinically confirmed cervical stenosis study subjects (N = 83).

In the present study, most of the clinically confirmed cases of cervical stenosis were noticed in vertebrae C4 (77.1%), C5 (78.3%), and C7 (73.5%) (Figure 1).

**Figure 2:**
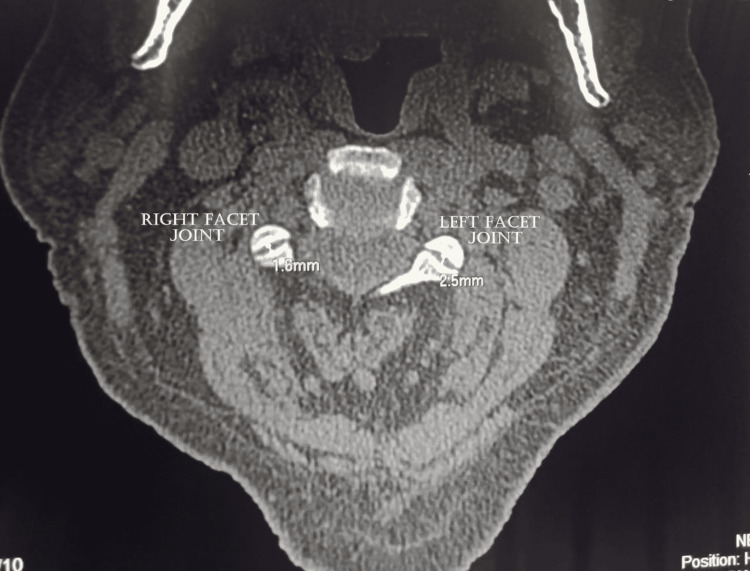
Axial CT image of the cervical spine showing measurements of right and left facet joints.

**Figure 3:**
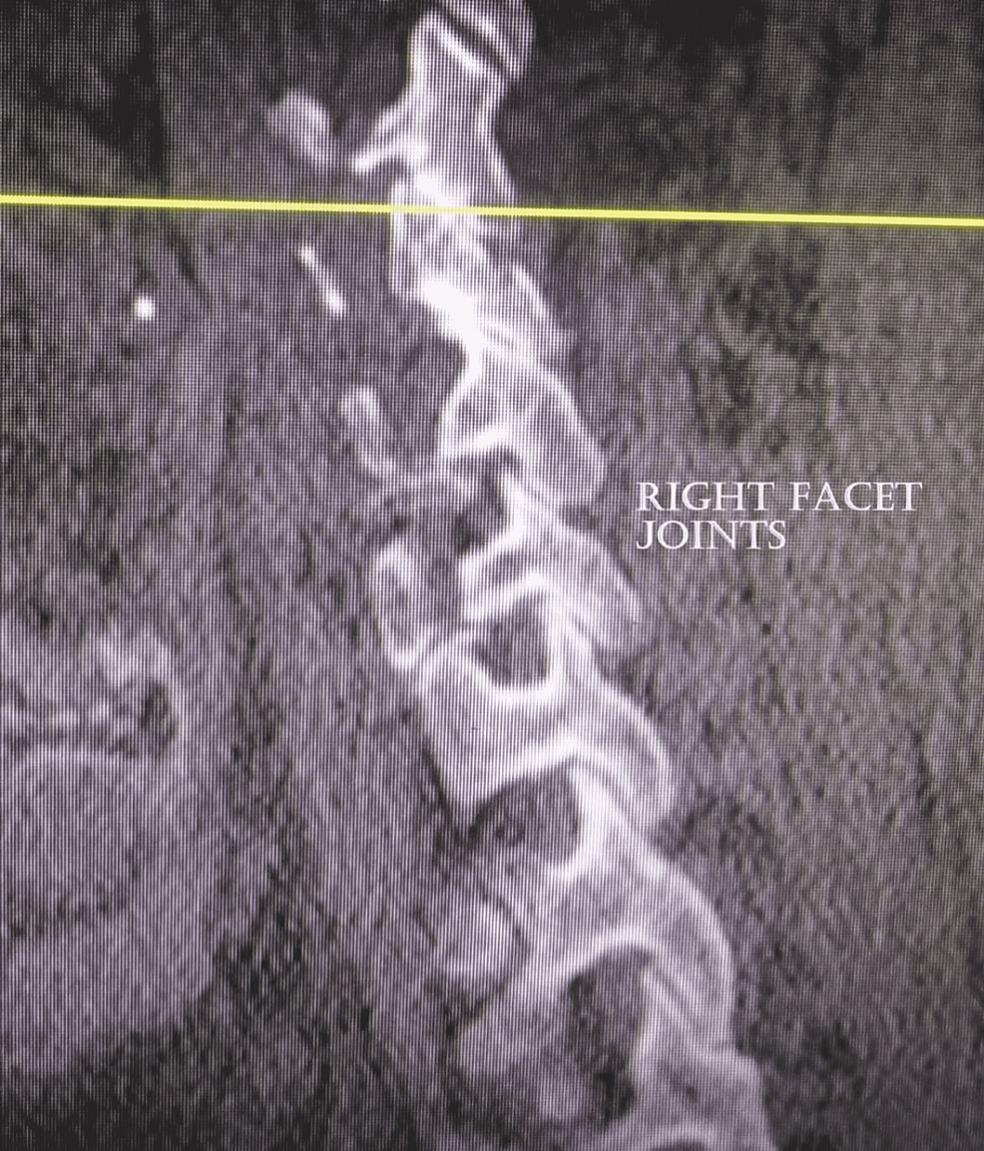
Sagittal CT image of the cervical spine showing right facet joints. Grade 2: joint space reduction, osteophytes formation, and hypertrophy.

The left facet joint evaluation showed the presence of joint space narrowing and the presence of osteophytes, the appearance of irregularity over the articular surface and second degree of facet joint degeneration, and moderate severity of left facet joint was observed among 50.6%, 38.5%, 49.4%, 37.3%, and 37.3% of study subjects, respectively (Figure 2). Similarly, the right facet joint evaluation showed the presence of joint space narrowing and the presence of osteophytes (Figure 3), the appearance of irregularity over the articular surface and second degree of facet joint degeneration, and moderate severity of right facet joint was observed among 48.2%, 36.1%, 45.8%, 33.7%, and 33.7% of study subjects, respectively (Table 4). Severe left and right facet joint degenerations were observed in 13.3% and 10.5% of study subjects, respectively.

**Table 4:** Multiple linear regression analysis of facet joint degeneration with independent variables among study subjects (N = 83). * R-squared = R^2^; # adjusted R-squared = aR^2^.

Dependent variable	Independent variables	R^2^*	aR^2#^	P-value
Right facet joint	Age	0.0119	−0.0388	0.9178
	Gender			
	Height			
	Weight			
Left facet joint	Age	0.0085	−0.0424	0.9548
	Gender			
	Height			
	Weight			

The multiple linear regression analysis of the degree of right facet joint degeneration was carried out with the independent variables (age, gender, height, and weight) and it was found to be not significant (R^2^ = 0.0119, aR^2 ^= −0.0388, p = 0.9178). Similarly, the multiple linear regression analysis of the degree of left facet joint degeneration was carried out with the independent variables (age, gender, height, and weight) and it was found to be not significant (R^2 ^= 0.0085, aR^2^ = −0.0424, p = 0.9548).

## Discussion

A total of 83 patients were enrolled in our study with an average age of 57.98 ± 7.63 years. This last variable is frequently linked to the degree of intensive work performed prior to the age of 20. Previous cross-sectional research has shown that even in asymptomatic healthy people, age-related alterations in the cervical spine are common. According to Freedman et al., patients who met the diagnostic criteria for myelopathy or myeloradiculopathy were enrolled in the "myelopathic" group with an average age of 64.4 ± 13.4 years [14].

In our study, 49.4% of males and 50.6% of females were enrolled. Kalichman et al. showed a high prevalence of FJOA in a community-based population (59.6% of males and 6.7% of females), which increased with age and reached 89.2% in individuals over 60 years old. Risk factors for FJOA include age, sex, spinal level, facet orientation (sagittal), and associated background of intervertebral disc degeneration. The correlation between degenerative changes in the FJOA and clinical back problems, on the other hand, is uncertain and a subject of considerable research [15].

Moreover, the T-VC was narrowest at the level of C3 (25.00 ± 1.13) and gradually increased at the level of C6 (25.18 ± 1.14) in this study. Zhang et al. included geometric parameters of normal cervical spinal canal including the sagittal and transverse diameter as well as the TR. Finally, they concluded that cervical spinal canal narrowing is associated with the extension of the cervical spine, gender, as well as ethnicity, and can lead to spinal cord injuries and neurological symptoms including neck pain, headache, weakness, and paresthesia [16-18].

The mean TR of cervical vertebrae C3 and C4 dropped gradually from C3 (0.78 ± 0.05) to C7 (0.76 ± 0.05) in this study. At the level of C4-C7, the mean TR remained constant. The origin of these declines is the severe pressing of the root of major nerves, which can be inside of the spinal canal, across the major zone of exits, or can be the VC’s outer part, resulting in pain of radicular origin. Similar compression of nerves can be caused by hypertrophied facets, focal osteophytes, facets rostrocaudal subluxations, and expanded capsules of effused facet joints [19]. Inflammation arising from facet structures can also migrate across the area of the myofascial, affecting numerous surrounding roots of nerves, or maybe even a swelled capsule of joints can put pressure on the surrounding root of nerves [20].

In this study, males had a large average distance between the spinal canal and the transverse foramen as compared to females. The right side had a longer mean distance between the spinal canal and the transverse foramen than the left side. This is consistent with our findings, which show that the left transverse foramen is larger than the right. The minimum level of distance between the spinal canal and the transverse foramen (dSC-TF) observed in male individuals was C5, whereas it was C4 in females. It usually causes mechanical neck pain, although it can also cause asymptomatic neck pain [21]. Previous works have shown that spinal pain is significantly generated by the facet joint, and certain therapies can bring relief to individuals presenting with pain and swelling of the facet joint. Worsening of symptoms is realized on extension while flexion is shown to bring relief and as such no discomfort radiating below the level of knees. Pain and deterioration have a weak association. Mechanical stress is increased at sagittal aspects that are more horizontal, generally C4-C5. Inflammation of the facet joint and associated soft tissues is becoming increasingly important in imaging investigations. Such inflammation is thought to be the source of pain that is confined locally (non-radiating). Excessive growth of bones, on the other hand, can lead to neuro-foraminal constriction and cause pain, which is radiating in nature [22].

In this study, the prevalence of spinal canal degeneration among patients with neck pain was noticed and demographic details were recorded. Of note, there was a high prevalence of joint space narrowing (50.6%), osteophytes (38.5%), irregularity of articular surface (49.4%), and second degree of facet joint degeneration (37.3%) in our study. Prabavathy et al. concluded the height of the pedicle, superior, and inferior articular processes decreased toward the lower cervical level. These results emphasize the importance of preoperative CT and conventional radiography of each patient in planning a surgical procedure and selecting the appropriate size of the instrument, thus avoiding possible postoperative complications related to the implant [23].

In our study, the frequency of neurologic symptoms was as follows: pain (98.8%), numbness (53.0%), tingling (25.3%), weakness (7.2%), and gait instability (6%). Despite the fact that physicians consult radiologists to assess the severity of FJOA, previous studies have found no association of low back pain symptoms with radiologically diagnosed cervical spinal canal degeneration [24]. So, it is a topic of research that needs more studies to provide facts on such association. FJOA is closely associated with DDD, and though discrete, but by function, it is a related disorder that affects components in the anterior spinal column.

Articular joint space narrows because of erosion along with sclerosis of the subchondral segment, hypertrophied bones and ligamentum flavum, and foramina invasion, which can lead to pain. Intraarticular gas, joint effusion, and spondylolisthesis are all secondary symptoms. Synovial cysts can form in the spinal canal or neuroforamen, extending posteriorly to the facet joint but also anterior. Joint traction during subluxation may cause gas to build up inside the joints (vacuum). These changes that are caused by osteoarthritis can be shown. There is a loss of disc height when the discs in your spine break down. This is called "rostrocaudal subluxation," and it puts your facet joints out of place [25]. When this happens, the facet joints are forced to bear a higher-than-normal share of the weight. Relaxed joints can lead to instability and subsequent arthrosis of facets [26]. In addition, disc herniation can alter the physiology of vertebral motion. Normally, the line for extension and flexion runs through disc space; however, a herniated disc can induce the line of articulation to run through these facet joints [27]. Flexions or extensions can be coupled to the swaying of posterior joints in this scenario, resulting in potential serious damages, such as fracture of the articular facet [28].

The prevalence of spinal stenosis was shown to be significantly different across groups of patients with and without neck pain. Neck pain and its consequences are a huge burden on the society, healthcare systems, and the economy of developed countries. There is little information available on the pathophysiology of neck pain, which is surprising given the high incidence of the complaints. Many practitioners commonly obtain radiography to establish a diagnosis as well as to provide confidence in clinical practice. Others say that radiography should only be used in subjects requiring invasive therapy and those who have evidence of potentially serious disorders because it can convey incorrect facts, cause undue stress, and lead to inaccurate treatment. Previous studies have revealed a higher proportion of asymptomatic subjects with spinal degeneration features, but there is no evidence to suggest a statistically significant association between such changes and the onset of neck pain in these patients [29,30].

## Conclusions

The articulations of the posterior arch of the vertebrae are known as facet joints. They are a vital component of the VC's structural stability. The superior and inner articular facets of the vertebrae are joined by these joints, which are encased in a fibrous capsule. The capsule of facet joints, spinous ligaments (inter and supra), and ligamentum flavum form the posterior ligamentous complex that holds vertebrae and facet joints in line. Neck pain is very common and has a huge impact on people's lives and employment. Imaging is used a lot to look at people who have specific back pain, even if they also have irradiating pain. The relationship between imaging-based anatomic anomalies, clinical history, and outcomes is still controversial. In certain circumstances, imaging investigations are unable to pinpoint the exact cause of neck pain. Neck pain is complex to diagnose and treat due to the sheer number as well as the diversity of probable pain triggers in the spinal lumbar region. Most of the studies on low back pain focused just on intervertebral discs; nevertheless, it has become abundantly clear that the facet joint (zygapophysial joint) plays a crucial influence. Spinal pain induced due to facets remains harder to diagnose. The facet joint may be the source of discomfort based on the patient's history and physical examination, but this cannot be confirmed.
